# In Murine Disseminated Candidiasis, Serum Amyloid P Component Inhibits Inflammation and C-Reactive Protein Potentiates Inflammation

**DOI:** 10.3390/pathogens15040436

**Published:** 2026-04-17

**Authors:** Stephen A. Klotz, Richard E. Sobonya, Peter N. Lipke

**Affiliations:** 1 Division of Infectious Diseases, Department of Medicine, University of Arizona, Tucson, AZ 85721, USA; 2 Department of Pathology, University of Arizona, Tucson, AZ 85721, USA; 3 Brooklyn College, City University of New York, Brooklyn, NY 11215, USA; plipke@brooklyn.cuny.edu

**Keywords:** disseminated candidiasis, pentraxins, C-reactive protein, serum amyloid P component, histology

## Abstract

*Candida albicans* is a ubiquitous commensal fungus that may be lethal once it gains access to the bloodstream, following a breach in protective barriers such as skin or gut lining. Intravenous injection of *C. albicans* (4.5 × 10^4^ yeasts/gm of mouse) leads reproducibly to systemic infection with a median survival of about 75 h. We studied the effects of two human innate immune effectors on the course of systemic infections. The soluble human pentraxin serum amyloid P component (hSAP) retards death in murine disseminated candidiasis. In contrast, another soluble pentraxin, human C-reactive protein (hCRP), hastens death. To examine the pathological basis for these differences, necropsies were performed, and the right kidney was removed for study. Candidiasis caused abundant collagen deposition (the precursor to fibrosis) and loss of contrast between the kidney medulla and cortex. Daily administration of subcutaneous hSAP following the intravenous injection of *C. albicans* preserved the discrete histological difference between cortex and medulla and lessened host collagen deposition. Yeasts and hyphae within abscesses were decorated with hSAP. Contrastingly, kidneys from animals administered *C. albicans* and hCRP showed extensive collagen deposition and loss of the boundary between the cortex and the medulla of the kidney. hCRP did not bind to fungi but bound to damaged tissue surrounding abscesses, leading to a more destructive infection with loss of tissue. Staining cells with antibodies to CD45 (to detect T-lymphocytes, myelocytes, monocytes, and macrophages) and antibodies to Ly-6G (neutrophils, and granulocytes) showed that hSAP retarded infiltration of inflammatory cells into diseased areas. The results are consistent with the hypothesis that early administration of hSAP represses the migration of inflammatory cells, dampens the production of collagen by fibroblasts, and dampens the overall immune response of the host to infection. In doing so, hSAP prolonged life, whereas hCRP facilitated the infectious process and hastened death.

## 1. Introduction

*Candida albicans* is an opportunistic pathogen, causing potentially fatal systemic disease if it gains access to the bloodstream after a breach in protective barriers such as the skin or gut lining. In both commensal and disease states, adherence is a crucial first step in colonization [1,2]. Among the *C. albicans* adhesins, the ALS gene family includes eight paralogs. Als1 and Als3 are expressed at high levels in biofilm formation and in systemic infections. These adhesins are among the proteins that form functional amyloids on the *C. albicans* surface, and the amyloids alter the course of pathogenesis [3,4]. Als5 (formerly Ala1) was the first *Candida* adhesin discovered, and it is important for commensal interactions [5]. For adherence to occur with Als1, Als3, or Als5p, a threonine-rich segment in the cell surface glycoprotein capable of undergoing amyloid formation must be present [6]. Functional amyloid nanodomains potentiate substrate adhesion through catch-bonding and facilitate fungal aggregation [7]. The Als amyloids also bind the soluble pattern recognition receptor (PRR) serum amyloid P component (SAP) [8]. This binding skews macrophages to the anti-inflammatory M2 state and modulates the immune response [4]. In human disease, *C. albicans* expresses surface functional amyloids, both in superficial infections and in deep abscesses [9]. We showed that other pathogenic fungi display surface amyloids during systemic infection as well [9]. Fungi with these surface amyloid nanodomains bind the soluble pattern recognition receptor (PRR) [10] serum amyloid P component (SAP) but not the related pentraxin, CRP [4]. SAP binds to all endogenous and exogenous amyloid in the human body, whether intra- or extra-cellular [11]. Consequently, we have been interested in the role of SAP and its effects on the pathogenesis of disseminated candidiasis.

SAP is a member of the pentraxin family of immune effectors. In humans, SAP is constitutively expressed, and when bound to pattern targets, it greatly affects innate immune response. Human SAP-coated *C. albicans* passivates the innate immune response to fungi: for example, macrophages that bind SAP-coated *C. albicans* are skewed toward the non-inflammatory M2 state. Such macrophages secrete the anti-inflammatory cytokine IL-10 and decrease secretion of the pro-inflammatory cytokines, TNF-a, IF-γ, IL-6, and IL-17 [4]. We have shown that, in mice challenged with a lethal dose of *C. albicans*, the administration of hSAP prolongs survival, whereas hCRP shortens lifespan [12]. Thus, these two PRRs have apparently opposite effects on the course of infection.

To confirm this hypothesis, we report here on the histology of kidneys from mice killed by disseminated candidiasis after treatment with SAP or CRP [12]. The results were consistent with an anti-inflammatory role for hSAP and a pro-inflammatory role for hCRP.

## 2. Materials and Methods

**Preparation of Fungi.** *C. albicans* SC5413 was maintained on YPD agar (RPI, Mount Pleasant, IL, USA). Yeasts were prepared for intravenous injection by removing a loopful of fungi from the plate and added to 50 mL of YPD liquid media (Life Technologies Corp., Carlsbad, CA, USA) maintained at room temperature (26 °C) in a shaking water bath. After 24 h growth, 1 mL of overnight growth was placed in 50 mL fresh YPD and cultured for 24 h. Yeasts were washed by centrifugation in TRIS buffer with Ca^2+^ pH 7.8. Yeast cells were counted, and dilutions were performed to achieve the desired yeast cell concentration for intravenous injection (4.5 × 10^4^ yeasts/gm of mouse).

**Experimental Protocol.** The role of SAP and CRP in a murine model of disseminated candidiasis was studied in the following manner. Male BALBcJ mice (20–25 gms) were administered 2 mg human SAP or CRP SQ, one day before the yeast administration (denoted as day −1). Yeast cells were administered intravenously on day 0, injected in the lateral tail vein (volumes of buffer were 90 to 130 µL). Additionally, SAP or CRP, 1 mg, was injected subcutaneously. Mice were checked 3 times/day and sacrificed when morbidly ill. Following death or sacrifice of animals by CO_2_ inhalation, the right kidney was removed, weighed and inspected visually, then placed in 10% formalin overnight followed by 70% ethanol. The right kidney is a reproducible target for adherence of intravenous *C. albicans* followed by infection [13]. Purified, authenticated, pathogen-free hSAP and hCRP were gifts from MB Pepys, University College, London.

**Animal Use.** Animal use protocol #19-587 was reviewed and approved by the University of Arizona Institutional Animal Care and Use Committee.

**Histology and Immunohistochemistry**. Tissue Acquisition Cellular/Molecular Analysis Share Resources at the University of Arizona performed the histology and immunohistochemistry. All kidney samples were embedded in paraffin, sectioned at 4 µm and stained with hematoxylin and eosin. A published protocol for the Masson Trichrome stain was followed [14]. Immunoreactivity was detected using the automatic platform Leica Bond RXm. Heat-induced antigen retrieval was done using the Bond Epitope Retrieval Solution 1 or 2 antibody (citrate-based pH6 Leica Biosystems, Catalog No. AR9961 or EDTA-based pH9, (Leica Biosystems, Catalog No. AR9640)). Immunoreactivity was detected using the Bond Polymer Refine Detection Kit (Leica Biosystems, Catalog No. DS9800). Since the polymer in the kit only detects rabbit and mouse IgG antibodies, a secondary antibody, rabbit anti-rat IgG (H+L) mouse absorbed (Vector, Catalog No. AI-4001) and rabbit anti-Goat IgG (H+L) mouse absorbed (Vector, Catalog No. AI-5000) were applied at 1:1000 and 1:3000, respectively, and incubated for 8 min at room temperature before the polymer application. See Table 1.

**Microscopy.** Microscopy was performed using the slide scanner, Leica Aperio AT2 (Leica Biosystems, Danvers, MA, USA) and photomicroscopy performed using the QuPath program [15]. Thin tissue slices were prepared and stained with the various tissue and antibody stains outlined in Table 1.

The end point for each mouse was death. The right kidney was removed, observed, and weighed prior to processing for histopathology. Each experiment consisted of 3 to 5 groups of 5 animals each; one set of five were controls (received only *C. albicans*); another set of 5 received hSAP and *C. albicans;* and another set of five received hCRP and *C. albicans*. Experiments were repeated a minimum of three times. All animals died of renal failure, an invariable accompaniment of intravenous injection of yeasts [13]. The photomicrographs are representative of 5 animals at death from each experimental group.

## 3. Results

**Preliminary Comments.** We focused solely on the histology of the mouse kidney. The kidney is the most reproducible target for adherence and infection following intravenous administration of yeast cells [12]. The intravenous injection of yeasts was followed with subcutaneous injection of human pentraxins. SAP and CRP serum levels differ in man and mouse. Although SAP is a constitutive glycoprotein in man, remaining constant from day to day with a mean concentration in men of ~32 mg/L and in women ~21 mg/L [11]. However, in the mouse, SAP behaves as the acute phase reactant, and CRP is constitutive. In humans, CRP is an acute phase reactant, rising from <1 mg/L to as much as 500 mg/L [11]. Therefore, its measurement is a valuable clinical tool, providing a measure of the severity of inflammation or infection [11]. The present results complement a prior report by us on the survival of mice given *C. albicans* intravenously, with or without subcutaneous hSAP or hCRP [12]. For all photomicrographs, the following applies: the end point for each mouse was death and the right kidney was removed and processed for histopathology. Each experiment consisted of three to five groups of five animals each; one set of five were controls (received only *C. albicans*); another set of five received hSAP and *C. albicans;* and another set of five received hCRP and *C. albicans*. Experiments were repeated a minimum of three times. All animals died of renal failure, an invariable accompaniment of intravenous injection of yeasts [13]. The photomicrographs are representative of five animals at death from each experimental group.

**Figure 1:**
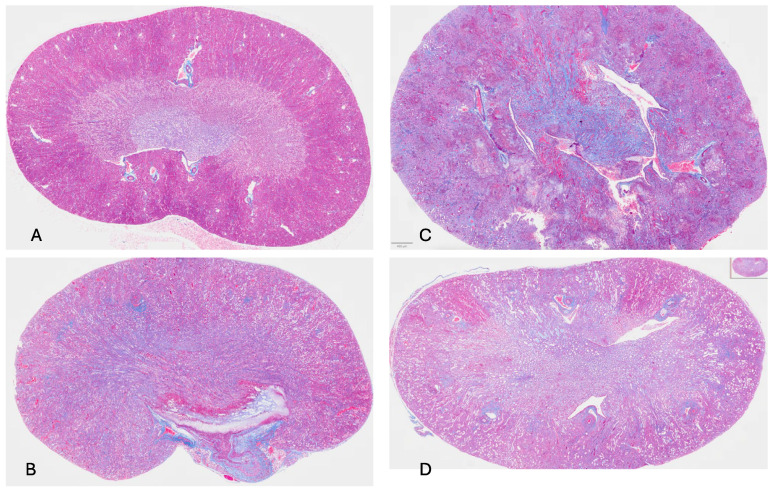
(**A**) Uninfected mouse right kidney (trichrome stain; 40×); no pentraxin was administered; the trichrome stain demonstrates the sharp distinction between the medulla (the central part of the kidney) and the cortical region of the kidney. (**B**) Right kidney from mouse given *C. albicans* only (trichrome; 40×); changes detectable 2 days after receiving *C. albicans* yeast cells intravenously. The distinction between medulla and cortex was lost and collagen deposition was extensive in the medulla and cortex (blue hue denotes collagen). (**C**) Right kidney from mouse given *C. albicans* intravenously and hCRP daily SQ. *C. albicans* was evident as a stringy, pinkish-red-stained infiltrate especially prominent in the cortex and medullary regions (trichrome stain; 40×). (**D**) Right kidney from mouse given *C. albicans* intravenously and hSAP daily (trichrome stain; 40×). Note that the characteristic histological difference between the cortex and medulla was maintained even though fungi (stringy pinkish material) were present in the medulla and less so in the medulla; the amount of collagen deposition was less than that in Figure 1B or Figure 1C.

**Damage to Kidney Following Intravenous Administration of *C. albicans* and Daily SQ hSAP or hCRP.** Blood filtration in the kidney occurs across basement membranes within the glomeruli, located in the cortex of the kidney. Urine collects and drains within adjacent tubules and then flows to collecting ducts found in the medulla of the kidney. Following intravenous administration of *C. albicans* to mice, blood cultures remained sterile for days [13]. Nevertheless, by day 2, kidney invasion was extensive, as demonstrated in Figure 1. In uninfected mice, the kidney, when stained with trichrome, displays a sharp contrast between the cortex and medulla (Figure 1A). In response to *C. albicans,* infection fibroblasts laid down collagen, particularly in the medulla and parts of the cortex (Figure 1B). These characteristics were exaggerated in mice injected SQ with hCRP and fungi (Figure 1C). In this circumstance, the kidney was swollen and lost its normal bean shape. There was abundant collagen deposition as well as *C. albicans* proliferation throughout the organ. Figure 1D shows the kidney from a mouse administered with *C. albicans* and daily hSAP. Surprisingly, the delineation between the medulla and cortex remained. There was reduced collagen deposition compared to the infected kidney (Figure 1B). Fungi are evident, particularly in the cortex of the kidney (scarlet red). Administration of hSAP spared much of the inflammatory destruction of the kidney as well as reduced collagen deposition, features that are seen most dramatically with the administration of hCRP and *C. albicans* alone (Figure 1C).

**Figure 2:**
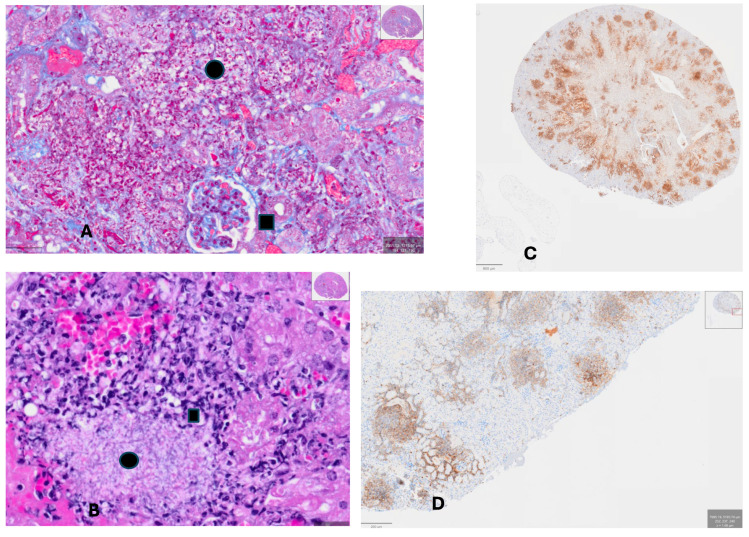
(**A**) Kidney of mouse that received *C. albicans* and hCRP. Trichrome stain demonstrates a glomerulus (black square) with proliferation of collagen and development of an abscess (pink yeasts and hyphae visible, black dot); there was a paucity of normal-appearing kidney parenchyma (~400×). (**B**) Trichrome stain of same kidney shows yeast and hyphae at the edge of the abscess (black square) with pink staining of fungi in the abscess proper (black dot, ~400×). (**C**) Same kidney of mouse given *C. albicans* and hCRP stained with anti-LyG-6 (staining neutrophils, monocytes and granulocytes; 40×); (**D**) same kidney of mouse given *C. albicans* and hCRP stained with anti-CD45 which stains leukocytes; (40×).

**Histology of Kidney from hCRP-Treated Mouse along with *C. albicans***. Figure 2A,B show developing abscesses of a mouse administered with *C. albicans* and daily hCRP. There was extensive proliferation of fungi (black circle) and fibrosis (blue hue) within an abscess near a glomerulus (black square) in Figure 2A, and an abscess (black circle) surrounded by inflammatory cells (black square) in Figure 2B. Figure 2C shows the same CRP-treated kidney stained with anti-Ly-G6 antibody, showing the antigen expressed on bone marrow-derived cells (e.g., myelocytes and macrophages). Figure 2D shows staining with anti-CD45 antibody which stains leukocytes, demonstrating marked infiltration of the kidney parenchyma of the medulla and cortex tissue with these inflammatory cells, especially adjacent to or within abscesses. hCRP treatment was associated with exuberant fungal growth and was permissive of inflammatory cell migration into the infected tissue.

**Figure 3:**
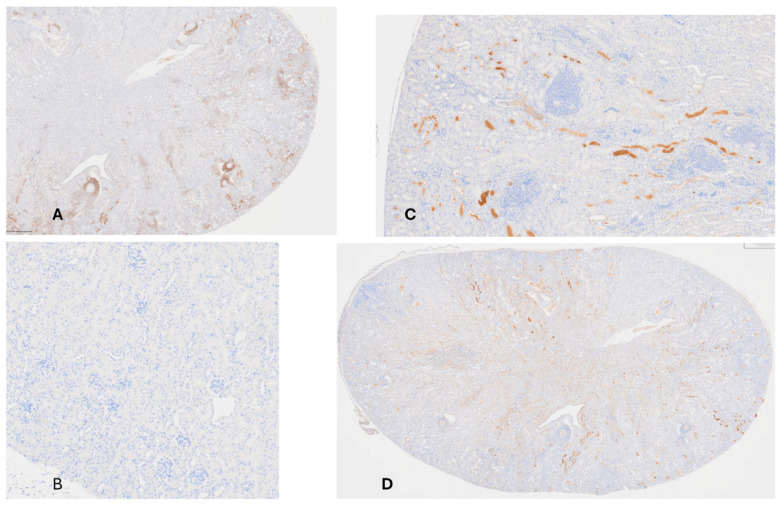
(**A**) Kidney of mouse given *C. albicans* and hSAP stained with anti-CD45. There is a muted invasion of leucocytes into inflamed/infected areas throughout the kidney at sites of abscesses (compare to Figure 2D) (40×). (**B**) Uninfected mouse kidney stained with anti-CD3e (stains all T cells) showing no uptake in cortex or medulla C (100×). (**C**) Kidney of mouse given *C. albicans* alone stained with anti-CD3e showing concentration of staining in glomeruli and their tubules and collecting ducts; there is no staining in adjacent abscesses (100×); (**D**) Kidney of mouse given *C. albicans* and hSAP and stained with anti-CD3e (same animal as Figure 3A stained with a different antibody); diffuse uptake in cortex and medulla indicating the presence of T cells throughout the kidney (as opposed to spotty areas shown in Figure 3C) (40×).

**Histology of Kidney from hSAP-Treated Mouse.** Figure 3A demonstrates that mice had a greatly diminished presence of leukocytes (stained by anti-CD45) in infected areas compared to the mouse given hCRP (cf. Figure 2D). T-lymphocyte distribution was faint and widespread, not concentrated around abscesses. Figure 3B–D show staining with anti-CD3e antibody. In the uninfected kidney (Figure 3B), there is homogeneous distribution of T-lymphocytes; in Figure 3C, uptake of anti-CD3e staining T cells is concentrated at the portal of entry of the fungus in the glomeruli and their tubules and collecting ducts extending into the medulla; Figure 3D shows widespread distribution of T-lymphocytes, perhaps greatest in the medulla for a mouse given *C. albicans* and hSAP. Compared to mice given *C. albicans* and hCRP, it is apparent that hSAP-treated mice have a muted cellular host response to infection.

**Figure 4:**
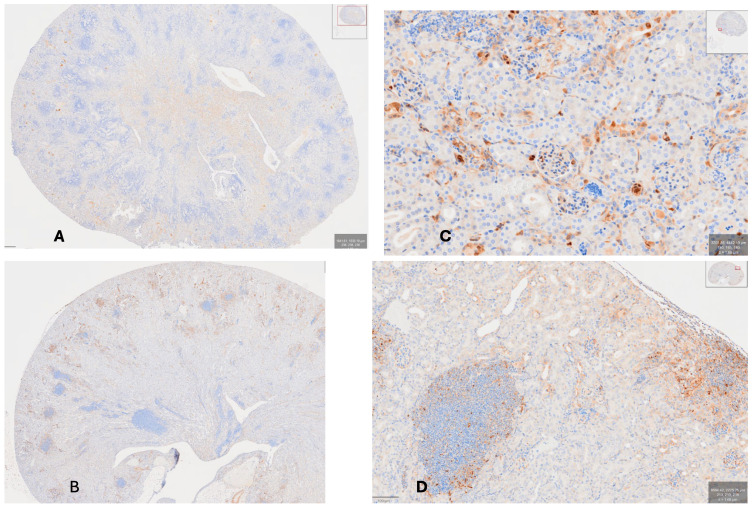
(**A**) Kidney of mouse given C. albicans and hCRP stained with anti-CD3e (same animal as Figure 2C stained with different antibody) showing the distribution of T cells in the medulla and cortex with an un-countable number of developing abscesses and do not contain any T cells (40×); (**B**) Kidney of mouse given *C. albicans* and hCRP stained with anti-CRP-1C (40×); (**C**) kidney of mouse given *C. albicans* and hSAP stained with anti-amyloid P (400×) showing staining of SAP to yeasts and hyphae on the periphery of a well-developed abscess; 400×; (**D**) staining with anti-amyloid P showing fungi scattered within an abscess (lower left) and a developing abscess near the cortical surface (on right of photograph).

**hCRP Binds to Damaged Kidney Tissue Whereas, hSAP Binds to Fungi**. Figure 4A shows the distribution of T cells in a mouse given *C. albicans* and hCRP with widespread development of abscesses. Figure 4B shows that the binding of hCRP occurs to damaged tissue surrounding abscess development, whereas hSAP (Figure 4C,D) binds to tissues with intact remnants of basement membrane as well as to yeasts and hyphae, especially at the outer edge of a large abscess (Figure 4D).

## 4. Discussion

*Candida albicans,* once disseminated in the human body, remains a highly lethal disease despite appropriate antifungal therapy [16]. We show here that SQ hSAP preserved kidney histology and concomitantly reduced infiltration of immune cells in an animal model of disseminated candidiasis. Thus, in this mouse model of disseminated candidiasis, SAP injections early in the infectious process ameliorated disease [12]. SAP reduces inflammatory cytokine production, upregulates IL-10 and binds to DCSign [17]. This sequence of events reduces the number of circulating fibrocytes and, thus, post infectious fibrosis. Exacerbation of the infectious process seen in disseminated candidiasis is similar to findings in the COVID-19 pneumonia, where the cytokine storm is a major contributor to the morbidity and mortality of hospitalized patients [18]. Fibrosis of pulmonary tissue due to infection is part of the normal inflammatory response to infection. When the response is dysregulated, as it often is in very ill patients with COVID-19, and occasionally in disseminated candidiasis, fibrosis due to collagen deposition is often the deleterious residue. We have discovered that SAP administration, when added early in the disease process, prolongs the survival of mice with disseminated candidiasis [12]. Nouresadeghi et al. found the opposite to be true in bacterial sepsis [19]. They found that SAP bound to bacteria and, in doing so, inhibited phagocytosis, which led to a lethal outcome for mice. We found that *C. albicans* binding SAP reduced the inflammatory response and enhanced survival [4,12]. In line with the salutary effect of SAP in infections, recombinant SAP was administered to patients for years, which resulted in amelioration and retardation of idiopathic pulmonary fibrosis [20].

Recently, Karhadkar et al. studied the COVID-19 pneumonia in a mouse model and found that recombinant SAP ameliorated the inflammatory response of the host, reducing cytokine production and fibrosis in the lung [21]. These results are similar to the results of our murine candidiasis study. Exacerbation of the infectious process seen in disseminated candidiasis is similar to findings in the COVID-19 pneumonia where the cytokine storm is a major contributor to the morbidity and mortality of hospitalized patients [18]. Fibrosis of pulmonary tissue due to infection is part of the normal inflammatory response to infection. When the response is dysregulated, as it often is in patients with COVID-19, and occasionally in disseminated candidiasis, fibrosis due to collagen deposition is often the deleterious residue. Interestingly, a study of COVID-19 patients in Intensive Care Units revealed that SAP blood levels had a negative association with fibrosis, indicating that high SAP levels corresponded with less fibrosis [18]. SAP thus appears to trigger an anti-inflammatory response and decreased tissue damage in several disorders. For example, it reduces keloid formation in individuals subject to the dysregulation of fibrocytes in wound healing [22]. Pending further study, perhaps employing immunomodulators [23], like SAP, judiciously in cases with dysregulated host immune responses, such as what occurs in COVID-19 and disseminated candidiasis (along with the standard agents such as antivirals and antifungals), might achieve better outcomes.

Our findings with CRP in the disseminated candidiasis model deserves further comment as well. The SQ hCRP in this animal model exacerbates the inflammatory response to *C. albicans* infections and led to pathological loss of kidney architecture and the additional infiltration of inflammatory markers and immune cells. Although purified hCRP is harmless when injected into humans [11], the protein, when bound to damaged tissue, can accelerate destruction. Consequently, removal of high levels of CRP associated with inflammation and infections shows some promise of disease improvement. For example, apheresis of CRP has been studied with patients with myocardial infarction [24]. Investigators found clinical improvement and less myocardial damage [24]. Other methods of reducing CRP post myocardial infarction include protein inhibitors of CRP that block its ability to bind to autologous ligands [9,25].

## Figures and Tables

**Table 1:** Stains and antibodies utilized in this investigation.

Stain	Purpose of Stain	How *Candida albicans* Appears with This Stain
Hematoxylin and Eosin (H&E) stain	To compare the normal histology of mouse kidney with mice infected with *C. albicans* intravenously; abscess formation is easily distinguished	Hyphae usually a light blue whereas yeast cells are light blue to pink
Trichrome	Demonstrates collagen (precursor to fibrosis) which stains blue; muscle fibers, cytoplasm, and keratin stain red, and nuclei stain black	*Candida albicans* stains pink
Antibody * (company/number)	**Host species of antibody. Conjugation/dilution if applicable**	**Reacts to:**
Abcam; ab211631	Unconjugated rabbit polyclonal to CRP-1—C-terminal; 1:400 dilution	Human/mouse CRP
Bethyl; Cat#IHC00613T	Unconjugated goat polyclonal antibody to C Reactive Protein; 1:800 dilution	Human CRP
Biocare Medical Cat#PP132AA	Unconjugated rabbit polyclonal antibody to amyloid P; incubated as prepared	Human SAP
Abcam; ab300481	Unconjugated Rabbit monoclonal [EPR26467-123] to SAP97; 1:3000 dilution	Human/mouse SAP
Biolegend; Antibody#362701 Clone APA1	Monoclonal to purified anti-human/mouse CD3ε (activation epitope); 1:200 dilution	Human/mouse CD3ε
Biolegend Antibody#103101 Clone 20-F11	Rat monoclonal to mouse CD45; 1:200	Mouse CD45
Purified Antibody#127602 Clone 1A8	Rat monoclonal to mouse Ly-6G; 1:400	Mouse Ly-6G

* The quality and specificity of all antibodies conformed to the Tissue Acquisition Cellular/Molecular Analysis Share Resources at the University of Arizona.

## Data Availability

The original contributions presented in this study are included in the article. Further inquiries can be directed to the corresponding author.
